# Detection method has independent prognostic significance in the PLCO lung screening trial

**DOI:** 10.1038/s41598-023-40415-y

**Published:** 2023-08-17

**Authors:** James P. Long, Yu Shen

**Affiliations:** Department of Biostatistics, The University of Texas MD Anderson Cancer Center, Houston, USA

**Keywords:** Cancer epidemiology, Non-small-cell lung cancer, Cancer epidemiology

## Abstract

Prognostic models in cancer use patient demographic and tumor characteristics to predict survival and dynamic disease prognosis. Past work in breast cancer has shown that cancer detection method, screen-detected or symptom-detected, has prognostic significance. We investigate this phenomenon in the lung component of the Prostate, Lung, Colorectal, and Ovarian (PLCO) screening trial. Patients were randomized to intervention, receiving four annual chest x-rays (CXRs), or to control, receiving usual care. Patients were followed for a total of approximately 13 years. In PLCO, lung cancer detection method has independent prognostic value exceeding that of variables commonly used in lung cancer prognostic models, including sex, histology, and age. Results are robust to cohort selection and type of predictive model. These results imply that detection method should be considered when developing prognostic models in lung cancer studies, and cancer registries should routinely collect cancer detection method.

## Introduction

Prognostic models are important for informing patients, caregivers, and clinicians about the likely disease course. Prognostic models often use patient demographic information (e.g., age, sex), clinical variables (e.g., cancer stage, histology), genetic information, and imaging features at the time of disease diagnosis. These models have been developed for many cancer types including glioblastoma multiforme (GBM)^1^, breast cancer^2^, and lung cancer^3–5^.

Many solid tumors are detected via screening such as colonoscopies for colorectal cancer, mammograms for breast cancer, and Pap tests (Pap smears) for cervical cancer^6–8^. In the near future, multi–cancer early detection tests (MECD) using minimally invasive procedures (e.g., blood draws) are expected to increase the fraction of cancers that are detected via screening^9,10^. Screen-detected cancers are known to have different characteristics than symptomatic cancers in general. In particular, screen-detected cancers are more likely to be found at an earlier stage (known as stage shift, which is a form of lead-time bias)^11–13^ and be slower growing (known as length bias)^14–16^.

Past studies have found that detection method can have prognostic significance on patient survival and disease recurrence. In a study of Finnish Cancer Registry patients diagnosed between 1991 and 1992, mammographically (screen) detected breast cancers were shown to have less chance of distant recurrence relative to tumors detected outside of screening, after controlling for cancer biological factors at diagnosis^17^. In three randomized screening trials, patients with screen-detected breast cancer (with mammography) were found to have better survival than symptom detected cancers, after controlling for stage^8^. This finding was further confirmed in a study of patients treated for breast cancer at the Netherlands Cancer Institute^18^, which found that detection method remained a significant prognostic factor after controlling for patient age, tumor size, grade, and hormone status, among other variables. A study of patients with Ductal Carcinoma In–Situ (DCIS) found that patients with mammographically detected tumors were less likely to develop invasive disease and had lower all-cause mortality^19^.

The reason why detection method has prognostic significance is not completely known and potentially consists of lead-time and length biases inherent in screening programs, as well as true survival benefit achieved due to effective treatment following an early diagnosis of cancer. Detection method may be a proxy for intra-stage shift (among tumors of a given stage, screen-detected tumors may be earlier in their development, on average, than symptom-detected tumors). Moreover, detection method may be a proxy for growth rate of tumor, a variable of obvious prognostic value that is not easily measured, and hence is rarely used in prognostic models. Slower growing or even indolent tumors have better prognosis regardless of detection method (length bias). In this study, we assess the prognostic significance of cancer detection method in the lung component of the Prostate Lung Colorectal Ovarian (PLCO) trial^20^. To our knowledge this is the first study to assess the prognostic value of detection method in lung cancer.

**Figure 1:**
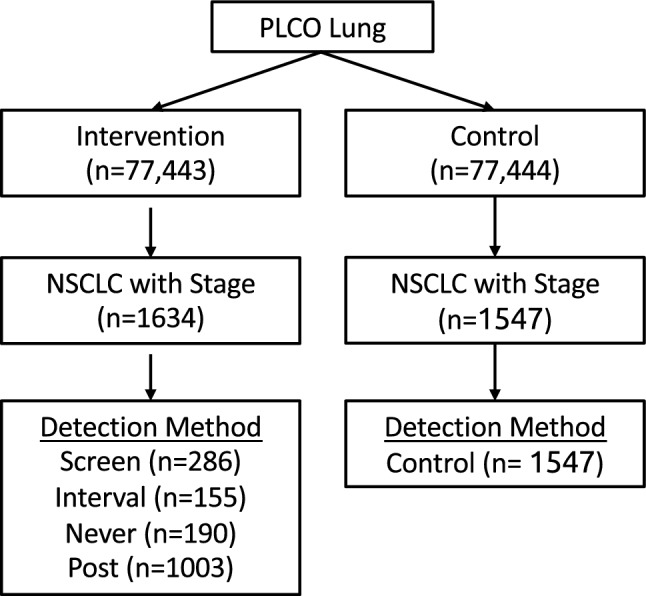
Summary of the lung component of the PLCO trial.

## Data sources and statistical methods

The study cohort is summarized in Fig. 1. The data contain participants aged 55–74 enrolled in PLCO over the years 1993 to 2001 and randomized to receive annual chest x-ray (CXR) ($n = 77,443$) or standard of care ($n = 77,444$). Annual screenings took place at years 0, 1, 2, and 3 in the intervention arm. Never smokers randomized after April 1995 were not offered the final screen (at year 3). Follow up extended to December 31, 2009. Annual screening with CXR was not found to reduce lung cancer mortality, compared with usual care^20^.

We consider non–small cell lung cancers (NSCLC) with stage at diagnosis available. Within the intervention arm (screening), 1634 NSCLC were detected, while 1547 were diagnosed in the control arm (no screening). NSCLC in the intervention arm were categorized into one of four groups for detection method: Screen (detected by CXR at the annual screening exams), Interval (detected between scheduled screening exams or up to 1 year after final screening), Never (participant was randomized to intervention but never participated in any screenings), and Post (cancer diagnosed more than 1 year after final screening). Data were obtained from the Cancer Data Access System (CDAS) under a material transfer agreement and IRB approved protocol (CDAS project PLCO-808).

For this study, the primary analyses (4-Year Cohort) was restricted to patients diagnosed during the first 4 years (screening period + 1 year follow-up) of the randomized trial when annual CXR was offered in the intervention arm. We merge together patients whose tumors were detected in the Never and Post group into an Other group because there are only ($n=6$) Post detected tumors occuring within 4 years after randomization. Note that cancers were diagnosed by symptoms in Interval, Other and Control groups. We also perform a secondary analyses using all patients diagnosed in the two arms over the extended follow-up of 13 years (denoted as Extended Cohort). This Extended Cohort includes the full set of n = 1003 Post tumors.

Prognostic variables on lung cancer cases were recorded including stage, age, sex, smoking history, histology at diagnosis, and time to death or censoring measured from diagnosis of NSCLC. We exclude small cell lung cancer (SCLC) from our analysis because prognostic models are generally constructed separately for SCLC and NSCLC and staging is determined differently for the two diseases^5^.

Survival curves are estimated using the Kaplan–Meier method^21^. Log-rank tests are used to compare survival curves. Fisher’s Exact Test with simulated p-values is used to assess dependence of categorical variables. The Cox Proportional Hazards (CoxPH) model and Random Survival Forests (RSF) are used to construct multivariate prognostic models^22,23^. RSF is an adaptation of Random Forests to right censored survival data. It is a non-parametric, ensemble learner. Permutation variable importance (VIMP) is used to assess the importance of each prognostic variable in RSF models^24,25^. Permutation VIMP is computed by selecting a variable, permuting its values randomly across the entire sample, and then computing the prediction error. The difference between the prediction error with the permuted variable and the prediction error with the original variable is computed. Higher differences imply the variable is contributing more to reducing the prediction error, and thus of higher prognostic value in the model. In the context of RSF, prediction error is defined as $1-C$ where *C* is Harrell’s Concordance-index.

Statistical analysis was performed in R version 4.1.1. The R packages gtsummary^26^, ggplot2^27^, survival^28^, survminer^29^, and randomForestSRC^23^ were used to fit models, make plots, and produce tables.

De-identified PLCO data was obtained from the Cancer Data Access System (CDAS) under approved project PLCO-808 (https://cdas.cancer.gov/approved-projects/3140/). Approval to analyze the data was given by the University of Texas MD Anderson Cancer Center (MDACC) Institutional Review Board (protocol 2021-0807, approved September 29, 2021). All analysis methods were carried out in accordance with relevant guidelines and regulations, following protocol specifications. Informed consent was waived by the MDACC Institutional Review Board.

## Results

### Detection method and prognosis

Table 1 summarizes baseline risk variables among patients by detection method. Screen, Interval, and Other represent cancers detected within the Intervention arm while Control represents cancers diagnosed in the Control arm. The p-values (Fisher’s exact test) assess dependence between detection method (columns) and each variable.

**Table 1:** Characteristics of lung cancers detected in PLCO.

Characteristic	Screen, N = 279	Interval, N = 148	Other, N = 81	Control, N = 426	p-value
Stage					< 0.001
Stage I	140 (50%)	39 (26%)	20 (25%)	101 (24%)	
Stage II	26 (9.3%)	10 (6.8%)	3 (3.7%)	44 (10%)	
Stage III	69 (25%)	42 (28%)	20 (25%)	134 (31%)	
Stage IV	44 (16%)	57 (39%)	38 (47%)	147 (35%)	
Age					0.5
≤ 59	58 (21%)	37 (25%)	11 (14%)	83 (19%)	
60-64	67 (24%)	35 (24%)	24 (30%)	125 (29%)	
65-69	98 (35%)	46 (31%)	28 (35%)	126 (30%)	
≥ 70	56 (20%)	30 (20%)	18 (22%)	92 (22%)	
Sex					0.5
Female	113 (41%)	59 (40%)	36 (44%)	157 (37%)	
Male	166 (59%)	89 (60%)	45 (56%)	269 (63%)	
Smoked					< 0.001
No	23 (8.2%)	8 (5.4%)	5 (6.2%)	29 (6.8%)	
Yes	256 (92%)	139 (94%)	63 (78%)	377 (88%)	
Unknown	0 (0%)	1 (0.7%)	13 (16%)	20 (4.7%)	
Histology					< 0.001
Adenocarcinoma	134 (48%)	58 (39%)	29 (36%)	189 (44%)	
Bronchiolo-alveolar	33 (12%)	10 (6.8%)	2 (2.5%)	20 (4.7%)	
Squamous cell	59 (21%)	33 (22%)	23 (28%)	109 (26%)	
Large cell	21 (7.5%)	10 (6.8%)	3 (3.7%)	26 (6.1%)	
Other NSC	6 (2.2%)	4 (2.7%)	2 (2.5%)	4 (0.9%)	
Carcinoma, NOS	21 (7.5%)	28 (19%)	21 (26%)	74 (17%)	
Other/Unknown	5 (1.8%)	5 (3.4%)	1 (1.2%)	4 (0.9%)	

n (%).

Fisher’s Exact Test for Count Data with simulated p-value (based on 2000 replicates).

There is evidence of stage shift when comparing the distribution of Stage I–IV cancers in the Screen group with the other three groups (p-value $< 0.001$). In particular, 16% of Screen cancers are Stage IV at diagnosis, while 35–47% of cancers detected in other groups are Stage IV at diagnosis. The Screen group is enriched for early stage, with 50% of screen-detected cancers being Stage I at diagnosis versus 24–26% for the symptom detected groups (Interval, Other, and Control).

The variable Smoked is distributed differently across the four groups ($p < 0.001$). This is due to a higher fraction of the Other group having Unknown smoking status. The Other group is composed mostly of individuals randomized to the Intervention arm who did not attend any screenings. Thus, it is not surprising that these individuals are less likely to respond to questionnaires regarding their Smoking status. Screen-detected tumors are more likely to be adenocarcinoma than symptom-detected cancers. This finding was noted in previous work and possibly reflects a higher sensitivity of screening to adenocarcinoma relative to other histologies^20^. Supplementary Table 1 summarizes variables among patients by cancer stage at time of diagnosis. Histology is associated with Stage with an enrichment of Carcinoma, NOS among Stage IV (24%) relative to the other three stages (6–17%).

**Figure 2:**
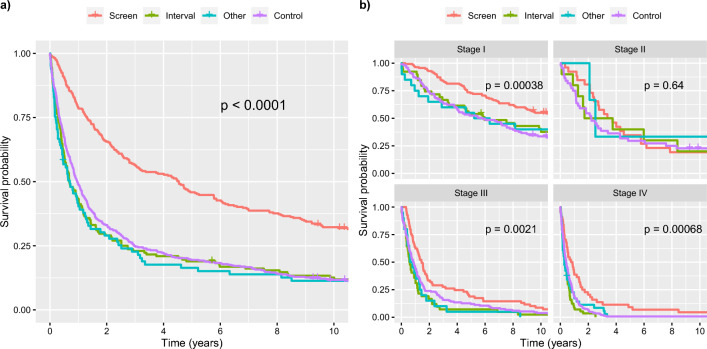
(**a**) Overall survival by detection method for NSCLC cases. (**b**) Overall survival by stage at diagnosis for each detection method. Detection method remains a significant prognostic variable after controlling for stage.

Figure 2a shows survival curves of time from diagnosis until death for each group. Screen-detected cancers have a substantially better prognosis by log-rank test (p-value $< 0.0001$). It is worth noting this finding does not imply that the screening intervention led to an actual benefit in survival time, as discussed earlier. Screening may detect cancers early while death times remain the same. This phenomenon, known as lead time bias, may also compound length bias.

Note also that the association between detection method and survival in univariate analysis does not necessitate that detection method will have independent prognostic value in a multivariate model after adjusting for tumor characteristics and other baseline risk factors. For example, the association between detection method and survival could be explained entirely by other variables such as stage (i.e., screen-detected cancers are more likely to be Stage I, which by itself has favorable prognosis). To investigate this issue, Figure 2b shows survival distributions by detection method within each stage at diagnosis. Stage at diagnosis is often the strongest predictor in multivariate prognostic models^5^. Detection method remains a strong predictor of survival within cancers detected at Stage I, III, and IV (p-values $< 0.005$). Patients with screen-detected stage I lung cancer have a longer median survival than those with stage I cancer diagnosed by symptoms (Screen median 11.52 years, Interval median 5.96 years, Other median 5.77 years, Control median 5.80 years). Lack of significance of detection method for Stage II may be due to the fact that relatively few lung cancers are Stage II at diagnosis (less than 10%). This leads to small samples sizes for comparing survival across detection methods. Supplementary Fig. 1 shows survival distributions by detection method within early stage (I/II) and late stage (III/IV) tumors. Patients with screen-detected tumors have better survival in both groups.

### Detection method in multivariate prognostic models

We further assess whether detection method has independent prognostic significance, beyond other commonly used variables (age, sex, stage, etc.) and the relative contribution of detection method to prognostic model performance. An existing lung cancer prognostic model, the Lung Cancer Prognostic Index (LCPI), was constructed using a cohort of $n = 695$ patients and subsequently validated on two patient cohorts selected from Australian metropolitan tertiary referral centers ($n = 479$ and $n = 284$)^5^. LCPI used a multivariate Cox Proportional Hazards model. We identified all variables in the LCPI model that are available in PLCO. We added the variable Detection indicating detection method to these variables and fit a CoxPH model to predict survival.

**Table 2:** Multivariate Cox PH model to predict overall survival following diagnosis with lung cancer.

Characteristic	log(HR)	95% CI	p value
Detection			
Screen	–	–	
Interval	0.61	0.39, 0.83	< 0.001
Other	0.46	0.19, 0.74	0.001
Control	0.43	0.26, 0.60	< 0.001
Stage			
Stage I	–	–	
Stage II	0.50	0.24, 0.77	< 0.001
Stage III	1.3	1.1, 1.5	< 0.001
Stage IV	2.0	1.8, 2.3	< 0.001
Age			
≤ 59	–	–	
60-64	0.28	0.07, 0.49	0.008
65-69	0.36	0.16, 0.57	< 0.001
≥ 70	0.71	0.49, 0.93	< 0.001
Sex			
Female	–	–	
Male	0.17	0.02, 0.31	0.024
Smoked			
No	–	–	
Yes	0.35	0.06, 0.65	0.020
Unknown	0.37	− 0.10, 0.83	0.12
Histology			
Adenocarcinoma	–	–	
Bronchiolo-alveolar	− 0.54	− 0.85, − 0.23	<0.001
Squamous cell	0.13	− 0.05, 0.31	0.2
Large cell	− 0.22	− 0.51, 0.07	0.13
Other NSC	− 0.22	− 0.76, 0.32	0.4
Carcinoma, NOS	0.15	− 0.05, 0.35	0.14
Other/Unknown	0.17	− 0.35, 0.70	0.5

HR, hazard ratio; CI, confidence interval.

Table 2 summarizes parameter estimates, confidence intervals, and p-values for the model. Detection is a highly significant prognostic variable (p-values for each level are $< 0.05$). The log hazard ratio of Screen to other detection methods (Interval, Other, and Control) ranges from 0.43 to 0.61. These estimated log hazard ratios are larger in absolute size than log hazard ratios for Sex and Smoked variables. Given all other variables being equal, a screen-detected Stage II tumor and an interval-detected Stage I tumor have similar prognosis. These findings show that detection method has high independent prognostic value.

Model coefficients for the prognostic variables agree well with what has been published in the literature. Hazard ratios increase with higher cancer stage at diagnosis, male sex, higher age, and smoking. The Cox model presented here reproduces findings from studies that found that bronchioloalveolar carcinomas have better prognosis than most NSCLC histological subtypes^30^. Note that the bronchioloalveolar classification has since been superseded by a new adenocarcinoma classification system^31^. Finally, the model obtains a Harrell’s C-Index of 0.76. This matches closely with LCPI, which obtained a 0.74 C-index.

### Secondary analyses: cohort selection and model choice

We assessed the robustness of these findings to cohort selection and choice of predictive model. First, we included all patients diagnosed with NSCLC cancer at any point in the active screening intervention period, as well as the extended follow-up of the trial. This is referred to as the Extended Cohort. Our primary analysis, restricted to subjects diagnosed within 4 years of randomization, is referred to as the 4-Year Cohort.

Supplementary Table 2 summarizes baseline risk variables among patients by detection method in the Extended Cohort. As expected, there is a large increase in the number of Other and Control cancers, relative to the 4-Year Cohort. Recall that Other includes individuals with cancers diagnosed in the Intervention arm more than 1 year after screening ended. For example, in the 4-Year Cohort there are $n = 426$ cancers diagnosed in the Control arm, while in the Extended Cohort there are $n = 1547$. Supplementary Fig. 2 displays Kaplan–Meier survival curves by detection method for the Extended Cohort. Detection remains a significant prognostic factor both in univariate analysis and after stratifying by stage. Supplementary Table 3 displays coefficient estimates, confidence intervals, and p-values from a CoxPH fit on the Extended cohort. Detection is a strong, independent prognostic variable in the model, with estimated log hazard ratio of Interval to Screen of 0.62 ($p<0.001$), similar to the results of the 4-Year Cohort (log HR of 0.61).

**Table 3:** Permutation variable importance in random survival forests models.

	4-Year cohort	Extended cohort
Stage	0.17631	0.16060
Detection	0.01440	0.00425
Age	0.00671	0.00601
Histology	0.00382	0.00713
Sex	0.00085	0.00311
Smoked	0.00064	− 0.00012

Detection is the second most important variable, after Stage, in the 4-Year Cohort. In the Extended Cohort, Detection is more important than both Sex and Smoked. These results show that method of detection has independent prognostic value in models other than CoxPH.

Next we considered robustness with respect to choice of the predictive model. While the CoxPH model is the most used regression model for right censored data, it assumes proportional hazards across time for different values of the covariates and that covariates have a linear effect on the log hazard function^32^.

To check whether our conclusions hold for other statistical models, we fit Random Survival Forests (RSF) to the 4-Year Cohort and the Extended Cohort, using the same variables as in the CoxPH model in Table 2^23^. The RSF model computes a measure of variable importance (VIMP). See the “Data sources and statistical methods” section for details on how to compute VIMP. Table 3 displays VIMP for the 4-Year Cohort and Extended Cohort, ordered by importance in the 4-Year Cohort (larger VIMP implies higher variable importance). As expected, Stage is the most important variable in the RSF models for both cohorts. In the 4-Year Cohort, Detection is the second most important variable relative to other baseline risk factors. In the Extended cohort, Detection is more important than Sex and Smoked. These findings confirm that detection method has independent prognostic value for models other than the Cox model.

## Discussion

We found that detection method has high independent prognostic value in NSCLC prognostic models beyond stage shift using data from the lung component of the PLCO screening trial. NSCLC patients with screen-detected tumors have better prognosis than those diagnosed by other detection methods. Results were robust to cohort selection and statistical modeling approach. This phenomenon has been noted before in breast cancer.

This study’s findings do not imply benefit of CXR screening on NSCLC patients’ survival. Lung cancer mortality at 13 years post randomization was found to be similar between the Intervention and Control groups^20^. Past work suggests two possibilities for why detection method has prognostic significance: (a) screen-detected tumors exhibit a within-stage shift where a screen-detected stage X tumor is (on average) at an earlier phase of the disease progress than a stage X tumor detected by symptoms (within stage shift), and (b) selection bias, where screen-detected tumors tend to be slower growing and hence less aggressive, but are more likely to be detected by periodic screening exams. Under the second possibility, detection method can be viewed as a proxy for the tumor growth rate, a variable with clear prognostic importance, which is not easily measured and typically not available for use in prognostic models. It is possible that both reasons contribute mutually to the prognostic significance of detection method. Note that randomized controlled trials may evaluate the mortality benefit of screening intervention properly, but they cannot remove the lead-time nor length (selection) bias as these are inherent in screening intervention.

Our study has several limitations. First, PLCO did not collect all variables used in the LCPI model. For example, the LCPI model used presence of actionable mutation and patient Eastern Cooperative Oncology Group (ECOG) status. Our conclusions about the independent prognostic value of detection method could be weakened or changed if variables such as presence of actionable mutations were included in CoxPH or RSF models.

Second, since the biological mechanism behind why detection method is a significant prognostic variable is not completely evident, results may not generalize to other lung cancer cohorts in which different screening interventions are used. For example, the National Lung Screening Trial (NLST) studied the effect of low-dose CT screening on lung cancer mortality^6^. Recently developed MECD use circulating cell-free DNA (cfDNA) to detect cancer^10^. Tumors detected with low-dose CT and cfDNA may not have the same properties as tumors detected with CXR, such as slow growth rate. It is of interest to explore the prognostic value of the LDCT screening using the NLST trial data in a future study.

It is likely that in the future a larger share of cancers will be detected via screening either by regular image or blood exams. In the area of lung cancer, NLST showed that low-dose CT scans reduce lung cancer mortality by 20% among smokers^6^. As a result, regular screening is now recommended for this cohort. More generally, MECD tests are now available to consumers.

Our findings imply that cancer detection method should be considered when constructing prognostic models. At present, detection method is not as widely collected as other variables used in prognostic models, such as age, sex, and tumor characteristics. Screening trials and cancer registries should collect cancer detection method, as it may be an important variable with prognostic value not captured by other measures.

### Supplementary Information

Supplementary Information.

## Data Availability

Data: De-identified PLCO data was obtained from the Cancer Data Access System (CDAS) under approved project PLCO-808 (https://cdas.cancer.gov/approved-projects/3140/). Approval to analyze the data was given by the University of Texas MD Anderson Cancer Center (MDACC) Institutional Review Board (protocol 2021-0807, approved September 29, 2021). All analysis methods were carried out in accordance with relevant guidelines and regulations, following protocol specifications. Informed consent was waived by the MDACC Institutional Review Board. Software: Computational codes for reproducing the figures and tables in the work are available at https://github.com/longjp/plco-lung-detection-method.
