# Supplementary material for: Detection method has independent prognostic significance in the PLCO lung screening trial
Source: Sci Rep. 2023 Aug 17;13:13382. doi: 10.1038/s41598-023-40415-y (PMC10435538; doi:10.1038/s41598-023-40415-y)
Supplement: Supplementary file 1 — Supplementary Information. [file 41598_2023_40415_MOESM1_ESM.pdf]

Supplementary Material to: Detection Method Has Independent Prognostic Significance in the PLCO Lung Screening Trial

James P. Long and Yu Shen

June 22, 2023

Supplementary Table 1: Variables distributions by Stage at Detection.

**Characteristic**	**Stage I**, N = 300	**Stage II**, N = 83	**Stage III**, N = 265	**Stage IV**, N = 286	**p-value**
Detection					<0.001
Screen	140 (47%)	26 (31%)	69 (26%)	44 (15%)	
Interval	39 (13%)	10 (12%)	42 (16%)	57 (20%)	
Other	20 (6.7%)	3 (3.6%)	20 (7.5%)	38 (13%)	
Control	101 (34%)	44 (53%)	134 (51%)	147 (51%)	
Age					0.7
<=59	60 (20%)	22 (27%)	50 (19%)	57 (20%)	
60-64	75 (25%)	20 (24%)	69 (26%)	87 (30%)	
65-69	95 (32%)	28 (34%)	87 (33%)	88 (31%)	
>=70	70 (23%)	13 (16%)	59 (22%)	54 (19%)	
Sex					0.2
Female	130 (43%)	32 (39%)	92 (35%)	111 (39%)	
Male	170 (57%)	51 (61%)	173 (65%)	175 (61%)	
Smoked					0.7
No	24 (8.0%)	5 (6.0%)	20 (7.5%)	16 (5.6%)	
Yes	267 (89%)	76 (92%)	237 (89%)	255 (89%)	
Unknown	9 (3.0%)	2 (2.4%)	8 (3.0%)	15 (5.2%)	
Histology					<0.001
Adenocarcinoma	133 (44%)	26 (31%)	109 (41%)	142 (50%)	
Bronchiolo-alveolar	42 (14%)	4 (4.8%)	11 (4.2%)	8 (2.8%)	
Squamous cell	76 (25%)	33 (40%)	71 (27%)	44 (15%)	
Large cell	20 (6.7%)	5 (6.0%)	18 (6.8%)	17 (5.9%)	
Other NSC	7 (2.3%)	2 (2.4%)	5 (1.9%)	2 (0.7%)	
Carcinoma, NOS	19 (6.3%)	12 (14%)	45 (17%)	68 (24%)	
Other/Unknown	3 (1.0%)	1 (1.2%)	6 (2.3%)	5 (1.7%)	

n (%)

Fisher's Exact Test for Count Data with simulated p-value(based on 2000 replicates)

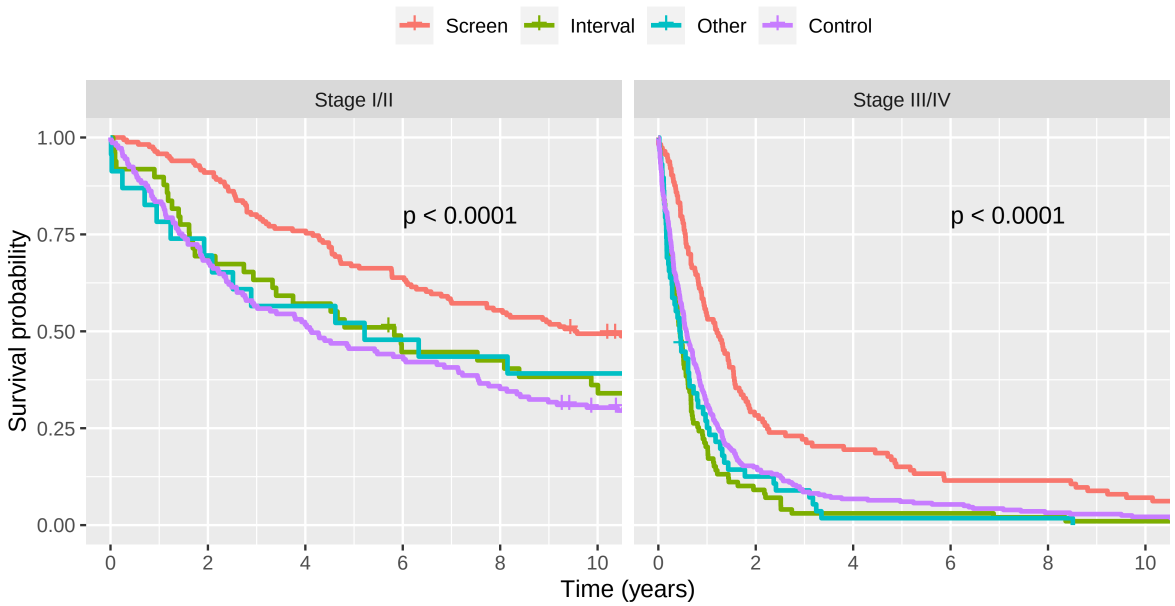

Supplementary Figure 1: Overall survival by stage at diagnosis (Stage I/II versus III/IV).

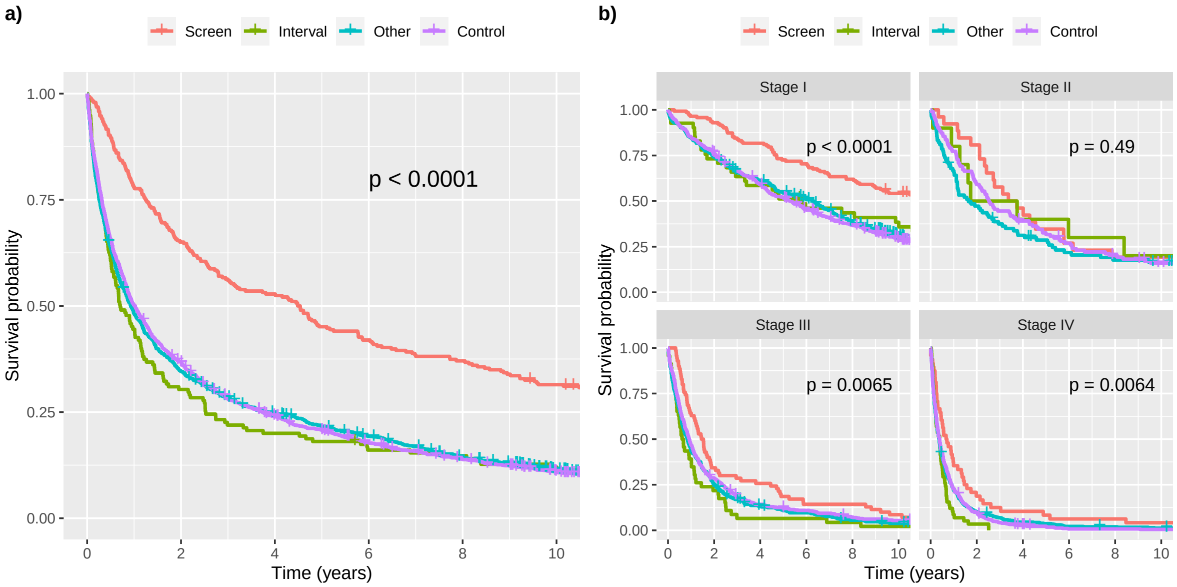

Supplementary Figure 2: a) Overall survival by detection method for NSCLC cases. b) Overall survival by stage at diagnosis for each detection method. Method of detection remains a significant prognostic variable after controlling for stage.

Supplementary Table 2: Characteristics of lung cancers detected in PLCO for Extended Cohort.

**Characteristic**	**Screen**, N = 286	**Interval**, N = 155	**Other**, N = 1,193	**Control**, N = 1,547	**p-value**
Stage					<0.001
Stage I	142 (50%)	41 (26%)	338 (28%)	428 (28%)	
Stage II	26 (9.1%)	10 (6.5%)	84 (7.0%)	110 (7.1%)	
Stage III	70 (24%)	46 (30%)	293 (25%)	406 (26%)	
Stage IV	48 (17%)	58 (37%)	478 (40%)	603 (39%)	
Age					0.080
<=59	59 (21%)	38 (25%)	221 (19%)	308 (20%)	
60-64	69 (24%)	36 (23%)	389 (33%)	487 (31%)	
65-69	100 (35%)	48 (31%)	358 (30%)	447 (29%)	
>=70	58 (20%)	33 (21%)	225 (19%)	305 (20%)	
Sex					>0.9
Female	116 (41%)	60 (39%)	464 (39%)	608 (39%)	
Male	170 (59%)	95 (61%)	729 (61%)	939 (61%)	
Smoked					<0.001
No	24 (8.4%)	8 (5.2%)	113 (9.5%)	118 (7.6%)	
Yes	261 (91%)	146 (94%)	1,043 (87%)	1,358 (88%)	
Unknown	1 (0.3%)	1 (0.6%)	37 (3.1%)	71 (4.6%)	
Histology					<0.001
Adenocarcinoma	137 (48%)	60 (39%)	475 (40%)	667 (43%)	
Bronchiolo-alveolar	33 (12%)	10 (6.5%)	58 (4.9%)	75 (4.8%)	
Squamous cell	62 (22%)	37 (24%)	289 (24%)	358 (23%)	
Large cell	21 (7.3%)	10 (6.5%)	45 (3.8%)	57 (3.7%)	
Other NSC	6 (2.1%)	4 (2.6%)	19 (1.6%)	26 (1.7%)	
Carcinoma, NOS	22 (7.7%)	29 (19%)	281 (24%)	337 (22%)	
Other/Unknown	5 (1.7%)	5 (3.2%)	26 (2.2%)	27 (1.7%)	

n (%)

Fisher's Exact Test for Count Data with simulated p-value(based on 2000 replicates)

Supplementary Table 3: Multivariate Cox PH model to predict overall survival following diagnosis with lung cancer. Model is fit on Extended Cohort.

**Characteristic**	**log(HR)**	**95% CI**	**p-value**
Detection			
Screen	—	—	
Interval	0.62	0.41, 0.82	<0.001
Other	0.46	0.32, 0.61	<0.001
Control	0.45	0.31, 0.60	<0.001
Stage			
Stage I	—	—	
Stage II	0.45	0.29, 0.61	<0.001
Stage III	1.1	1.0, 1.2	<0.001
Stage IV	1.8	1.7, 1.9	<0.001
Age			
<=59	—	—	
60-64	0.17	0.06, 0.28	0.003
65-69	0.37	0.26, 0.48	<0.001
>=70	0.66	0.54, 0.78	<0.001
Sex			
Female	—	—	
Male	0.27	0.19, 0.35	<0.001
Smoked			
No	—	—	
Yes	0.26	0.11, 0.40	<0.001
Unknown	0.44	0.20, 0.67	<0.001
Histology			
Adenocarcinoma	—	—	
Bronchiolo-alveolar	-0.63	-0.81, -0.44	<0.001
Squamous cell	-0.05	-0.14, 0.05	0.3
Large cell	-0.15	-0.34, 0.04	0.13
Other NSC	-0.17	-0.46, 0.11	0.2
Carcinoma, NOS	0.25	0.15, 0.35	<0.001
Other/Unknown	0.04	-0.22, 0.30	0.8

HR = Hazard Ratio, CI = Confidence Interval
